# Routine Karyotyping Reveals Frequent Mosaic Reciprocal Chromosome Translocations in Swine: Prevalence, Pedigree, and Litter Size

**DOI:** 10.1038/s41598-020-64134-w

**Published:** 2020-05-04

**Authors:** Samira Rezaei, Brendan Donaldson, Daniel A. F. Villagomez, Tamas Revay, Nicolas Mary, Daniela A. Grossi, W. Allan King

**Affiliations:** 1 Department of Biomedical Sciences, University of Guelph, Guelph, ON N1G 2W1 Canada; 2 Departamento de Produccion Animal, Universidad de Guadalajara, Zapopan, 44100 Mexico; 3 Alberta Children’s Hospital Research Institute (ACHRI), University of Calgary, Calgary, AB T2N 1N4 Canada; 4 UMR INRA-ENVT 444 Génétique Cellulaire, École Nationale Vétérinaire de Toulouse, 23 Chemin des Capelles - BP 87614, 31076 Toulouse, Cedex 3 France; 5 Fast Genetics, 8,4001 Millar Avenue, Saskatoon, SK S7K 2K6 Canada; 6 Karyotekk Inc. Box 363 OVC, University of Guelph, Guelph, ON N1G 2W1 Canada

**Keywords:** Biotechnology, Genetics

## Abstract

In the routine commercial karyotype analysis on 5,481 boars, we identified 32 carriers of mosaic reciprocal translocations, half of which were carrying a specific recurrent translocation, mos t(7;9). An additional 7 mosaic translocations were identified through lymphocyte karyotype analysis from parents and relatives of mosaic carriers (n = 45), a control group of non-carrier boars (n = 73), and a mitogen assessment study (n = 20), bringing the total number of mosaic carriers to 39 cases. Mosaic translocations in all carriers were recognized to be confined to hematopoietic cells as no translocations were identified in fibroblasts cells of the carriers. In addition, negative impact on reproduction was not observed as the fertility of the carriers and their relatives were comparable to breed averages, and cryptic mosaicism was not detected in the family tree. This paper presents the first study of mosaic reciprocal translocations identified in swine through routine screening practices on reproductively unproven breeding boars while presenting evidence that these type of chromosome abnormalities are not associated with any affected phenotype on the carrier animals. In addition, the detection of recurrent mosaic translocations in this study may emphasize the non-random nature of mosaic rearrangements in swine and the potential role of genomic elements in their formation.

## Introduction

Chromosome translocations in a mosaic state are rarely reported in domestic animal species. To date, over 200 constitutional chromosome rearrangements have been reported in the domestic pig^1^, while only four mosaic abnormalities are previously documented for this species^2,3^. The discrepancy in reports is mainly due to the fact that constitutional chromosome rearrangements are present in each individual cell, including germ cells, thus adversely affecting reproduction in otherwise phenotypically normal carriers^4–6^. Therefore, the primary focus of cytogenetic screening programs in domestic animal species is the identification of constitutional chromosome abnormalities, and the removal of carrier animals from the breeding population^7^. As such, cytogenetic screening programs are continually being performed on the domestic pig, which have allowed for accurate estimates of the prevalence and rate of formation for constitutional rearrangements in this species^6–8^. Between 0.5-1.5% of reproductively unproven young boars are estimated to be carriers of constitutional chromosome rearrangements, with reciprocal chromosome translocations being the most prevalent chromosome structural abnormality reported so far^1,6–9^.

Typically, routine karyotype analysis, which differentiates normal from abnormal chromosome constitutions, is based on the careful analysis of at least two optimal Giemsa banded karyotypes (GTG-banded), predicated on constitutional chromosome abnormalities being present in any single somatic cells^10,11^. As such, chromosome rearrangements present in a low-grade mosaic state may often be missed during routine cytogenetic screening^11–13^. Thus far, only four documented mosaic abnormalities in the domestic pig have been identified through investigation of specific boars. The first reported mosaic translocation for the domestic pig was a mos t(1;11), identified in a malformed stillborn piglet^2^. The other three mosaic translocations, a mos t(7;9), a mos t(7;18), and a mos t(9;18), were proposed to occur recurrently in 13 otherwise healthy pigs as a result of illegitimate recombination between the four genes that make up the porcine T-cell receptor (TCR)^3^. Beyond the description of these four mosaic chromosome rearrangements little is known about the general prevalence and characteristics of mosaic chromosome rearrangements in the domestic pig karyotype.

Over the past five years, we have conducted cytogenetic screening of over five thousand young boars that were reproductively unproven at the time of testing. Here, we report on the prevalence and the reproductive significance of somatic mosaic chromosome translocation in the domestic pig.

## Materials and Methods

### Cytogenetic screening of pig populations

Experimental protocols were approved by the University of Guelph’s Animal Care Committee, and carried out in accordance with the Canadian Council on Animal Care and the University of Guelph’s Animal Care Committee guidelines^14^. Peripheral blood samples were routinely collected from boars, aged approximately six months and raised at various Canadian farms, by experienced farm technicians or Canadian Food Inspection Agency veterinarians. These animals were raised under conventional husbandry confinement, which provided good animal health and welfare, thus the animals were not selected for research purposes. The whole blood samples were submitted to the Animal Health Laboratory of the University of Guelph for commercial genetic screening. Data from this analysis was provided for use in this study. Lymphocyte cultures from whole blood samples were set up according to standard cytogenetic protocols, as previously published^6,15^. For each animal twenty-five metaphases were captured using a camera mounted Leica microscope (Leica Camera AG, Germany), connected to a computer, with 10x and 100x objectives and saved on Openlab 5.02 software and a minimum of two optimal quality GTG-banded karyotypes were arranged at the level of 400 bands resolution via SmartType Software (Digital Scientific UK)^15^.

During the study, a total of 5,481 reproductively unproven young boars were cytogenetically screened in this way. If a chromosome rearrangement in a mosaic state was identified within the screened metaphases (typically 15), or in the first two optimal karyotypes, additional images were captured and a total of twenty-five metaphases were karyotyped for each carrier. In this study, karyotype analysis of 25 cells is referred to as “intensive” karyotype screening since routine screening involves assessment of 2-3 karyotypes. In addition, mosaicism over 9% is excluded with 90% confidence with a minimum 25 karyotypes^16^. In some cases were appropriate, to exclude mosaicism over 5% with 90% confidence 50 karyotypes were analyzed^16^.

### Pedigrees

When a boar was identified as a carrier of a chromosome rearrangement in a mosaic state, its sire, dam, and siblings were examined. In total, 45 relatives were available for sampling, for which twenty-five karyotypes were arranged and analyzed (Supplemental Table 1). In families where the sire, dam, or sibling of a carrier was also identified as carrying a chromosome rearrangement, a family tree of the affected pedigree was arranged showing the karyotype of each family member investigated.

### Control group

A group of 66 control animals was randomly selected from a pool of previously karyotyped breeding boars that were determined as chromosomally normal upon analysis of 2 routine karyotypes, and were unrelated to any carrier. In addition, 7 of the control animals had their sires available for karyotyping, analyzed in order to demonstrate sire-boar duo relationships. In total, 73 animals including the seven sire and boar pairs had an additional twenty-five karyotypes arranged.

### Effect of mitogen on the frequency of mosaic translocations

The primary mitogen in our routine karyotyping is Phytohemagglutinin (PHA; M from, Gibco, Grand Island, USA) which is a well-known T-cell mitogen^17,18^. In this study, in order to narrow down the B-cell or T-cell specificity of mosaic translocations in the blood, in addition to assessing the effect of mitogen on the frequency of mosaic translocations, lymphocyte chromosome preparations from 20 boars were chosen to undergo dual culture. Duplicate cultures were established simultaneously with either PHA or pokeweed mitogen (L8777, PWM from *Phytolacca americana*, Sigma-Aldrich, Saint Louise, USA). Intensive karyotype screening was performed to detect mosaic translocations in these two groups.

### Fibroblast culture

Ear or tail biopsy, where available from the breeders’ routine sampling, were collected from mosaic carrier boars (n = 10) and family members (n = 24). Fibroblast cultures were set up with some modifications according to the protocol by Stanyon and Galleni (1991)^17^. Intensive karyotype screening was performed on each fibroblast sample as described above.

### Statistical analysis on production parameters of rearrangement carrier and relatives

Student’s *t*-tests were performed to assess fertility based on two parameters: piglets alive after 24 hours and mummies. Fertility data from 47 animals: 4 mosaic carriers, 18 sires of mosaic carriers, 18 dams of mosaic carriers, and 7 sisters of mosaic carriers were compared to their corresponding breed averages. The number of piglets alive after 24 hours and mummies were recorded by the farms. For comparison the farms provided the breed averages for each metric.

## Results

Chromosome analysis was performed on the lymphocyte metaphase spreads from 5,529 pigs, consisting of 5,481 young boars and 45 relatives of mosaic carriers. Besides identification of a total of 53 constitutional chromosome abnormalities^1^, a total of 39 mosaic reciprocal translocation carriers were also identified (Table 1). All mosaic carriers demonstrated an average rate of mosaicism (4–8%), with the exception of case #29 which was observed to have a high level of mosaicism, with 60% of cells carrying identical mosaic translocations (Table 1).

**Table 1:** Mosaic translocation carriers in the Canadian swine population.

Carrier Number	Breed	Mosaic Translocation	Number of Cells Analysed	Mosaic Frequency/ 100 cells
1	Large White (Yorkshire)	t(7;9)(q24;q24)	25	4
2	Pietrain	t(7;9)(q24;q24)	25	4
3	Duroc	t(7;9)(q24;q24)	25	4
4	Landrace (French)	t(7;9)(q24;q24)	25	4
5	Landrace (Canadian)	t(7;9)(q24;q24)	25	4
6	Landrace (French)	t(7;9)(q24;q24)	25	4
7	Duroc	t(7;9)(q24;q24)	25	4
8	Yorkshire (French)	t(7;9)(q24;q24)	25	4
9	Duroc	t(7;9)(q24;q24)	50	4
10	Yorkshire (French)	t(7;9)(q24;q24)	25	4
11	Duroc	t(7;9)(q24;q24)	25	4
12	Duroc	t(7;9)(q24;q24)	25	4
13	Yorkshire (French)	t(7;9)(q24;q24)	50	2
14	Duroc	t(7;9)(q24;q24)	25	4
15	Duroc	t(7;9)(q24;q24)	25	4
16	Duroc	t(7;9)(q24,q24), t(3;13)(q21;q49)	25	8
17	Yorkshire (Canadian)	t(7;9)(q24;q24), t(6;7)(q21;q22)	50	4
18	Landrace	XX*/XY**; XY t(7;9)(q24;q24)	49	2
19	Duroc	t(7;18)(q22;q11)	25	4
20	Large White (Yorkshire)	t(7;18)(q22;q11)	25	4
21	Landrace	t(9;18)(q22;q11)	50	2
22	Duroc	t(3;7)(p15;q13)	25	4
23	Landrace (Canadian)	t(8;9)(q21;q24)	50	2
24	Landrace (French)	t(3;7)(q23;q26)	50	2
25	Landrace (French)	t(3;10)(q23;p13)	25	4
26	Yorkshire (French)	t(7;7)(q24;q15)	25	4
27	Yorkshire (French)	t(7;9)(q26,q22)	50	2
28	Duroc	t(2;8)(q23;q21)	25	4
29	Duroc	t(5;9)(q21;p22)	48	60
30	Yorkshire (French)	t(6;16)(p15;q21)	50	2
31	Duroc	t(7;9)(q15;q15)	25	4
32	Pietrain	t(7;13)(q22;q21)	25	4
33	Landrace (French)	t(9:13)(p32:q41)	50	2
34	Landrace (Canadian)	t(7;9)(q24;q24)	25	4
35	Yorkshire (French)	t(7;9)(q24;q24)	25	4
36	Duroc	t(7;9)(q24;q24)	25	4
37	Duroc	t(10;12)(q15;q11)	25	4
38	Yorkshire (Canadian)	t(9;18)(q22;q11)	25	4
39	Yorkshire (French)	t(5;7)(q21;q22)	25	4

*38XX 42/49 cells, **38XY 7/49 cells.

### Routine cytogenetic screening of young reproductively unproven boars

**Figure 1:**
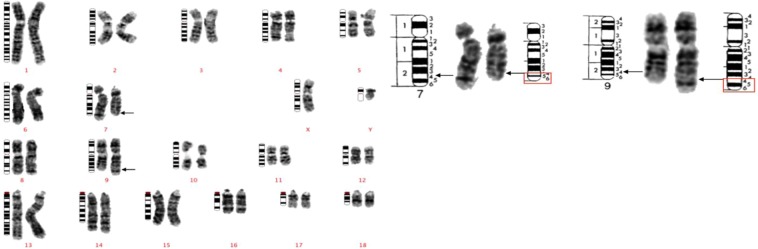
A GTG-banded karyotype representing the recurrent mos t(7;9). Arrows indicate the presumed breakpoints on the derivative chromosomes. Ideograms are placed on the left, normal chromosomes are placed in the middle, and their derivate chromosomes are placed on the right. On the left, the karyotype of case #5 is presented which is a Landrace boar diagnosed with the recurrent mos t(7;9) in the lymphocyte chromosome spread. On the right, the presumed breakpoints on chromosome 7 and chormosome 9 are compared to the ideogram.

**Figure 2:**
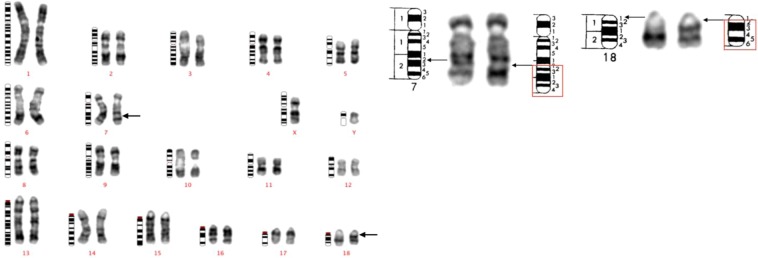
GTG-banded karyotype of case 19, a Duroc boar, representing the recurrent mos t(7;18). Ideograms are placed on the left, normal chromosomes are placed in the middle, and their derivate chromosomes are placed on the right. Arrows indicate the presumed breakpoints. On the left, is the GTG-banded karyotype of the metaphase containing the mos t(7;18) in a Duroc boar. On the right, is a detailed presentation of the breakpoints on chromosome 7 and chromosome 18 compared to the ideogram.

Under the routine screening procedure of 5,481 boars, we identified the presence of 32 unreported mosaic reciprocal translocations. Considering 2 optimal karyotypes per animal, mosaic translocations were identified in about 3 out of 1000 cells karyotyped. Interestingly, 18 of the carriers were diagnosed with a presumably identical reciprocal translocation, a recurrent mos t(7,9)(q24;q24) abnormality (Fig. 1). Additionally, two carriers of an observably identical t(7;18)(q22;q11) reciprocal translocation were identified in boars from Duroc and Yorkshire breeds (Case #19, Fig. 2 and case #20, Supplemental Fig. 1). Moreover, a carrier of a t(9;18)(q22;q11) in a mosaic state was identified in our routinely screened population (Case #21, Supplemental Fig. 2). Later we identified a presumably identical mos t(9;18) in the mitogen assessment group, making mos t(9;18) a recurrent translocation (Case #38, Supplemental Fig. 3). The boars carrying recurrent mosaic translocations were from various breeding populations (Table 2) and unrelated to one another. In addition, except for two boars, all the carriers were observed to have a single metaphase carrying the translocation, otherwise twenty-four metaphases with a normal karyotype complement were recorded for each animal.

**Table 2:** Breed and type of mosaicism involved in the routinely screened boar population.

Breed	Case Number	Duroc	Yorkshire	Landrace	Pietrain
**Recurrent mosaic t(7;9)**	1–18	8	5	4	1
**Recurrent mosaic t(7;18)**	19–20	1	1	—	—
**Recurrent mosaic t(9;18)**	21	—	—	1	—
**Non-recurrent mosaic carrier**	22–32	4	4	3	1
**Total Mosaic Carriers (n** = **32)**	1–32	13	10	7	2

The two boars that were revealed to have multiple distinct cell lines among the 25 metaphases karyotyped were cases #16 and #18. Carrier #16 showed three different cell lines among 25 metaphases karyotyped, with 23 cells having a normal chromosome constitution, and two cells each harbouring a unique translocation, a mos t(7;9)(q24;q24) in one, and a mos t(3;13)(q21;q49) in the other (Supplemental Fig. 4). While carrier #18 was described as a recurrent mosaic t(7;9)(q24;q24) and a chimera, the first of such kind in our screened population (Supplemental Fig. 5).

In the case of a Yorkshire boar (case #17), the recurrent mosaic translocation t(7;9) was identified through the routine screening of 2–3 karyotypes. Arranging additional karyotypes, bringing the total number to 25, did not reveal further abnormalities. However, a second blood sample was possible to obtain from the animal and upon analysis of a further 25 karyotypes (50 in total), a mosaic t(6;7)(q21;q22) was identified (Supplemental Fig. 6), otherwise, 48 metaphases with a normal karyotype were documented for this animal.

The remaining carriers (Supplemental Figs. 7 to 17), were all identified with unique, non-recurrent, mosaic translocations in 4–8% of their cells^16^ (Table 1).

### Family trees

**Figure 3:**
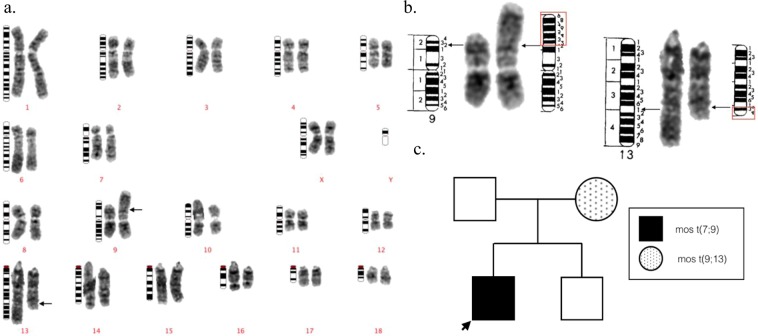
The GTG-banded karyotype of case 33, a Landrace dam of case 4, carrying a mos t(9;13). Ideograms are placed on the left, normal chromosomes are placed in the middle, and their derivate chromosomes are placed on the right. Arrows indicate the presumed breakpoints on derivative chromosomes. (**a**) The karyotype of the abnormal cell. (**b**) The detailed description of the breakpoints on the derivative chromosomes. (**c**) The pedigree of the case 4.

**Figure 4:**
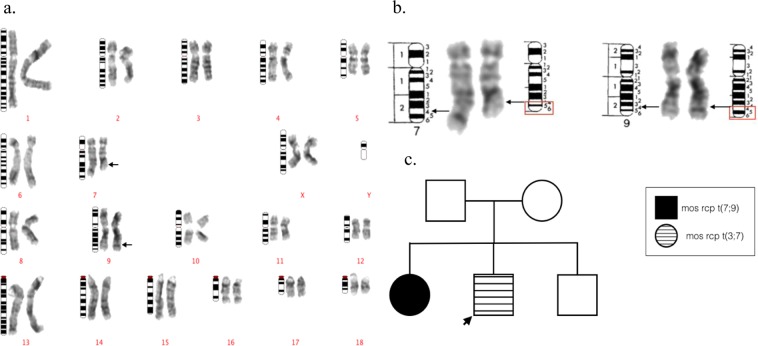
The GTG-banded karyotype case 34, the sister of case 24. Ideograms are placed on the left, normal chromosomes are placed in the middle, and their derivate chromosomes are placed on the right. Arrows indicate the presumed breakpoints on derivative chromosomes. (**a**) The karyotype of the abnormal cell mosaic t(7;9) in case #34. (**b**) The detailed description of the breakpoints on the derivative chromosomes. (**c**) The pedigree of case 24.

**Figure 5:**
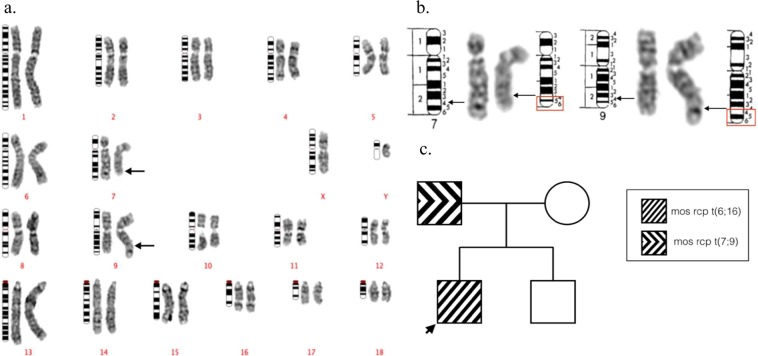
The GTG-banded karyotype case 35, the sire of case 30. Ideograms are placed on the left, normal chromosomes are placed in the middle, and their derivate chromosomes are placed on the right. Arrows indicate the presumed breakpoints on derivative chromosomes. (**a**) The karyotype of the abnormal cell identified as mos t(7;9) in case #35 (**b**) The detailed description of the breakpoints on the derivative chromosomes. (**c**) The pedigree of case 30.

Although mosaic translocations are presumably post-zygotic events and may not be inherited^23,24^, any available family members of the mosaic carrier boars (Cases #1–32) were cytogenetically analysed for chromosome abnormalities in order to establish any patterns of inheritance, or a genetic predisposition to any other type of genome instability. Intensive cytogenetic screening of 45 relatives of carrier boars led to the identification of 3 additional mosaic translocations (Supplemental Table 2). Each relative that was found to be a mosaic translocation carrier, had a different rearrangement from that of their relative (Case #33- Fig. 3, #34- Fig. 4, and #35- Fig. 5). Mosaic translocations were observed in relatives of rearrangement carriers at a rate of 2.6 per 1000 cells (Supplemental Table 3).

### Control study

Through our intensive cytogenetic screening of the relatives of mosaic rearrangements carriers, we identified a mos t(7;9) abnormality in the sire of carrier # 30, previously identified as having a normal chromosome constitution (Case #35- Fig. 5a). This observation led us to arrange and analyze additional karyotypes on 73 boars previously identified as normal. A recurrent mosaic t(7;9) was diagnosed in a Duroc boar (Case #36- Supplemental Fig. 18). Mosaic rearrangements were observed in control boars at a rate of 0.5 per 1000 cells.

### Effect of mitogen variability

To determine the effect of mitogen on the frequency of mosaic translocations, duplicate cultures with either PHA or PWM mitogen were established for 20 young reproductively unproven boars. Intensive karyotype screening led to detection of three mosaic translocations. Two mosaic translocations were identified in the PWM cultures, a mos t(10;12) in a Duroc boar (Case #37- Supplemental Fig. 19) and a mos t(5;7) in a Yorkshire boar (Case #39- Supplemental Fig. 20), and one mosaic t(9;18) in the PHA culture of a Landrace boar (Case #38- Supplemental Fig. 3). Mosaic translocations were observed at a rate of 4 per 1000 cells in PWM cultures and 2 per 1000 cell in the PHA cultures.

### Karyotype analysis of fibroblast chromosomes

To explore the tissue distribution of mosaic translocations, we analyzed the skin fibroblast chromosomes of 10 mosaic carriers and 10 relatives of mosaic carriers. In addition, 14 offspring from carrier #24 were available for tissue chromosome analysis. Fibroblast chromosome analysis did not reveal any mosaic chromosome translocations. However, an abnormal karyotype in a mosaic state in one offspring (Case #40, Supplemental Fig. 21) and a chimera abnormality in a stillborn were identified for which the peripheral blood was not available for further chromosome analysis (Supplemental Table 2).

### Analysis of reproduction based on piglets alive after 24 hours and mummies

As some mosaic rearrangements in humans are identified in individuals due to associated fertility problems^20–22^, we assessed the fertility of 4 mosaic carriers. The values of piglets alive after 24 hours and mummies, as measures of fertility, in carriers #24, #33, and #35 did not significantly differ from the average fertility of their corresponding breeds averages (p > 0.05; Student’s t-test) (Table 3). In carrier #34 the p-values for piglets alive after 24 hours (p = 0.01; Student’s t-test) and mummies (p = 0.02; Student’s t-test) were significantly lower than average, however only 2 litters were reported for this animal which may have skewed the results. In order to expand the examination of fertility we analysed the production parameters of 43 relatives of mosaic carriers and still found no significant difference compared to breed averages (p = 0.4631; Student’s t-test).

**Table 3:** Fertility of mosaic translocation carriers and their p-values compared to breed averages.

Case #	Carrier	Breed	Litter #	Average Alive After 24 Hours	Breed Average Alive After 24 Hours	p-value	Mummies	Breed Average Mummies	p-value
24	mos t(3;7)	L	5	11.2	12.12	0.72	0.4	0.34	0.81
33	mos t(9;13)	L	4	13	12.12	0.52	0.5	0.34	0.59
34	mos t(7;9)	L	2	8.5	12.12	0.01	0	0.34	0.02
35	mos t(7;9)	Y	13	13.07	12.09	0.48	0.53	0.36	0.41

## Discussion

According to our observations, mosaicism for balanced reciprocal chromosome translocation are underreported in the domestic pig. To date only 4 mosaic reciprocal chromosome translocations have been documented in the domestic pig^2,3^, in contrast to the over 200 total constitutional chromosomal rearrangements reported in the literature^1^. In this study we report a total of 39 mosaic chromosome translocations identified in the domestic pig through which we attempt to estimate the prevalence, possible tissue distribution, and interrogate the possible adverse biological effects of somatic chromosome mosaicism in this species.

In the routinely screened Canadian breeding boar population, the rate of mosaic chromosome translocation carriers was estimated at 0.58%. Mosaic translocations were identified in 32 young reproductively unproven boars, 21 of which were carriers of a recurrent mosaic event (Supplemental Table 1). Since the identification of non-recurrent somatic reciprocal translocations are unique to our study of the domestic pig, molecular mechanisms that arise the formation of these translocation events remain unknown in this species. Although in human it has been suggested that external factors such as UV radiation or internal mutagenic factors i.e. DNA repair mistakes may give rise to somatic variations^18,19^.

Each of the non-recurrent rearrangements were novel, and not previously reported by other studies, however the recurrent translocations identified in this study, mos t(7;9), mos t(7;18), and mos t(9;18) were previously investigated in a study by Musilova et al. [2014] in which the role of recurrent mosaic translocations in ageing as well as predisposition to cancer was investigated. Three breakpoint regions, (7q15.3-q21, 18q11.3-q12 and 9q21–22) where T-cell receptor (TCR) genes reside in the domestic pig, were investigated and the frequency of trans rearrangement were analyzed. The three recurrent translocations, mos t(7;9), mos t(7;18), and mos t(9;18) reportedly occur due to mistakes during V(D)J recombination in lymphocyte maturation at a rate of 0.18%, 0.22%, and 0.01%, respectively^3,20^. In the human genome, recurrent translocations between TCR genes in the lymphocyte of individuals have been identified to alter gene function and lead to the activation of proto-oncogenes^21,22^. Despite the presumably proximal and similar translocation breakpoints of recurrent translocations in our study to the TCR gene locations, and comparable frequencies between the two studies, further molecular analysis should be performed to confirm the breakpoints of recurrent mosaic translocations in this study in association with TCR genes.

Although non-recurrent rearrangements have not been reported in the literature, the breakpoints of some of these rearrangements are known to reside in unstable chromosome regions. Five of these mosaic rearrangements have breakpoints which overlap with cytogenetic bands reported to have common fragile sites (2q23, 6p15, 8q21, 12q11, 13q21, and 16q21)^23^. In addition, one non-recurrent mosaic rearrangements has a breakpoint, 16q21, which overlaps with an evolutionary breakpoint region^24^. It is however unclear whether these unstable genomic regions contributed to rearrangement at these positions.

Mosaicism in humans has also been associated with ageing^25,26^. A decline in the genome’s capacity to maintain its integrity, and an increase in somatic mutations have been suggested to account for the rise of mosaic abnormalities with age^19,25^. Despite such correlations, mosaic translocation carriers in our study were young animals with apparently normal phenotypical and physiological features. In addition, Musilova et al. [2014] did not find significant differences between the frequency of recurrent translocations in young and middle-aged pigs. Thus, ageing may not have factored into the development of mosaic translocations observed in peripheral blood of young boars.

Then, in order to ascertain the lymphocyte tissue specificity and the timing of mutation of the observed mosaic translocations in the young boars, it was necessary to analyze another tissue type for chromosome abnormalities^11,26^. Fibroblast chromosomes obtained from skin tissue are derived from ectoderm, another germ layer than mesoderm from which lymphocytes are developed^26^. Thus, the absence of mosaicism in fibroblast may indicate that mosaic translocations in the carriers are developed later in embryonic development or after birth and mosaicism may unlikely to be present in the germ cells^26^. Intensive fibroblast chromosomes analysis on skin tissue of 10 carriers and 10 relatives of mosaic carriers did not reveal any chromosome abnormality. It can thus be speculated that the mosaic abnormalities observed in the carrier animals were confined to hematopoietic cells, having occurred during lymphocyte maturation, with no indication that other tissues were affected.

Furthermore, litter size of four mosaic translocation carriers were compared to their corresponding breed averages, since in human mosaic translocations are often identified in individuals due to associated fertility problems or the presence of disease^20–22^. We found no significant deviation from the herd averages in the number of piglets alive after 24 hr or the number of mummies, in the litters of mosaic translocation carriers (Table 3). Although fibroblast chromosome analysis on 14 offspring of mos t(3;7) carrier #23, did reveal an abnormal mosaic karyotype in a live offspring and a sex chromosome chimera in a stillborn, inheritance of the mos t(3;7) was not detected. As mosaic abnormalities were not inherited by the offspring, and a decrease in litter size was not observed, mosaicism in the carriers may be regarded as somatic and confined to hematopoietic cells.

Somatic mosaicism was further confirmed by pedigree and family tree analysis of karyotyped relatives of carriers. Although 15.7% of mosaic carriers had relatives that were mosaic translocation carriers themselves, cryptic germline mosaicism may not be presumed since mosaic translocations in relatives were distinct from that of the pedigree. Although, a predisposition to developing mosaicism may be suspected since 6.25% of relatives of mosaic translocation carriers were mosaic carriers themselves compared to the 0.58% observed in the routinely screened boars and 1.36% in the control boars. In other words, the rate of mosaicism amongst groups were quite different, 2.6 per 1000 cells in family members, and 0.5 per 1000 cells in control boars, thus the prevalence of identifying mosaic translocations were 4.5 times higher in cells analyzed from family members than in cells from control animals. Despite the P-value of 0.14 (p > 0.1) comparing frequency between the latter groups, we suggest the possibility of some genetic factors predisposing to somatic chromosome mosaicism in the pigs.

This study is the first to report the prevalence of mosaic chromosome translocations in the routinely screened young reproductively unproven Canadian boar population. The frequency of mosaic translocations in young reproductively unproven boars is 0.58%. Additionally, considering the results of our intensive cytogenetic screening, the total prevalence of mosaic rearrangements carriers in the population was 0.7%. Mosaic translocations are presumably confined to hematopoietic cells and cryptic germline mosaicism was not observed as the pedigrees completed did not reveal similar translocations. However, predisposition to mosaicism was speculated due to the presence of mosaicism in 6.25% of the relatives.

Recurrent mosaic translocations in this study comprised half of all the mosaic translocations identified, all of which were presumably in or nearby TCR genes^3^. Thus, further molecular and genome analysis should be performed to consolidate the genomic sequences of breakpoint regions in order to discover the elements involved in forming the illegitimate joining of chromosome segments and identify the patterns of predisposition for ectopic genome exchanges. It has been suggested that mosaic chromosome rearrangements may play a role in the genome diversity of carriers, and may have implications in the generation of disease^18^. However, the exact role that mosaic chromosome rearrangements play is not fully understood. The domestic pig is one of the most studied large mammal species, and has the largest number of known chromosome rearrangements in domestic species^27^. Thus, the domestic pig is well positioned for the study of the prevalence and characteristics of chromosome rearrangements.

## Supplementary information

Supplementary information.
