# Supplementary material for: Routine Karyotyping Reveals Frequent Mosaic Reciprocal Chromosome Translocations in Swine: Prevalence, Pedigree, and Litter Size
Source: Sci Rep. 2020 May 4;10:7471. doi: 10.1038/s41598-020-64134-w (PMC7198520; doi:10.1038/s41598-020-64134-w)
Supplement: Supplementary file 1 — Supplementary information. [file 41598_2020_64134_MOESM1_ESM.docx]

**Routine Karyotyping Reveals Frequent Mosaic Reciprocal Chromosome Translocations in Swine: Prevalence, Pedigree, and Litter Size**

Samira Rezaei ^1^,Brendan Donaldson ^1^, Daniel A. F. Villagomez ^2^, Tamas Revay ^1,3^, Nicolas Mary ^4^, Daniela A. Grossi ^5,^ and W. Allan King ^1,6,*^

^1^ Department of Biomedical Sciences, University of Guelph, Guelph ON N1G 2W1, Canada

^2^ Departamento de Produccion Animal, Universidad de Guadalajara, Zapopan 44100, Mexico;

^3^ Alberta Children’s Hospital Research Institute (ACHRI), University of Calgary, Calgary, AB T2N 1N4, Canada

^4^ UMR INRA-ENVT 444 Génétique cellulaire, École nationale vétérinaire de Toulouse, 23 chemin des Capelles - BP 87614, 31076 Toulouse cedex 3, France

^5^ Fast Genetics, 8,4001 Millar Avenue, Saskatoon, SK S7K 2K6, Canada

^6^ Karyotekk Inc. Box 363 OVC, University of Guelph, Guelph, ON N1G 2W1, Canada

* Corresponding author. Email: waking@ovc.uoguelph.ca

**Supplementary Table 1. Mosaic carriers and the relatives for which peripheral blood was analyzed. * Animals for which both peripheral blood and skin tissue were analyzed. ** Animals for which only skin tissue was analyzed.**

| Case # | Sire | dam | Brother | Sister | Offspring | Uncle | Grandsire | Great Grandsire |
| --- | --- | --- | --- | --- | --- | --- | --- | --- |
| 4 | X | 33* | X | - | - | - | - | - |
| 5 | X | X | - | - | - | - | - | - |
| 6 | X | X | - | - | - | - | - | - |
| 8* | X | - | X** | X*^(n=4)^ | - | - | - | - |
| 9* | X | - | X* | X*^(n=2)^ | - | - | - | - |
| 10* | X | - | - | - | - | - | - | - |
| 13* | X | - | - | - | - | - | - | - |
| 15 | - | - | X | - | - | - | - | - |
| 17* | - | X | - | - | - | - | - | - |
| 18* | X | - | - | - | - | - | - | - |
| 21 | X | X | - | - | - | - | - | - |
| 23* | - | X | X | - | - | - | - | - |
| 24 | X | X | X* | 34* | X**^(n=14)^ | - | - | - |
| 25* | X | X | - | - | - | X ^(n=2)^ | - | X |
| 26 | X | X* | X | - | - | - | - | - |
| 27 | X | X | - | - | - | - | - | - |
| 29 | - | X | - | - | - | - | - | - |
| 30 | 35 | X | - | X | - | - | - | - |
| 32 | X | - | - | - | - | - | X | - |

Supplementary Table 2. Karyotype results of the relatives of mosaic carriers. Only the examined relative’s karyotypes are shown. Relatives with abnormal karyotypes are shown in bold.

| Case # | Carrier Karyotype | Sire Karyotype | Dam Karyotype | Brother Karyotype | Sister Karyotype | Other | % Affected of Examined Relatives |
| --- | --- | --- | --- | --- | --- | --- | --- |
| 4 | mos t(7;9) | Normal | **mos t(9;13)** | Normal | - | - | 33% |
| 5 | mos t(7;9) | Normal | Normal | - | - | - | 0% |
| 6 | mos t(7;9) | Normal | Normal | - | - | - | 0% |
| 8 | mos t(7;9) | Normal | - | Normal | Normal (n=4) | - | 0% |
| 9 | mos t(7;9) | Normal | - | Normal | Normal (n=2) | - | 0% |
| 10 | mos t(7;9) | Normal | - | - | - | - | 0% |
| 13 | mos t(7;9) | Normal | - | - | - | - | 0% |
| 15 | mos t(7;9) | - | - | Normal | - | - | 0% |
| 17 | mos t(7;9);(6;7) | - | Normal | - | - | - | 0% |
| 18 | mos t(7;9),XX/XY | Normal | - | - | - | - | 0% |
| 21 | Mos t(9;18) | Normal | Normal | - | - | - | 0% |
| 23 | mos t(8;9) | - | Normal | Normal | - | - | 0% |
| 24 | mos t(3;7) | Normal | Normal | Normal | **mos t(7;9)** | - | 25% |
| 25 | mos t(3;10) | Normal | Normal | - | - | Normal (n=3) | 0% |
| 26 | mos t(7;7) | Normal | Normal | Normal | - | - | 0% |
| 27 | mos t(7;9)* | Normal | Normal | - | - | - | 0% |
| 29 | mos(5;9) | - | Normal | - | - | - | 0% |
| 30 | mos t(6;16) | **mos t(7;9)** | Normal | - | Normal | - | 33% |
| 32 | mos t(7;13) | Normal | - | - | - | Normal | 0% |

**Supplementary Table 3. Frequency of mosaic translocations per 1000 cells in various groups.**

|  | Boars | Relatives | Control Group | Mitogen Assessment |
| --- | --- | --- | --- | --- |
| Population | 5,481 | 45 | 73 | 20 |
| Mosaic Translocations | 32 | 3 | 1 | 3 |
| Number of Karyotypes | 10,962 | 1,125 | 1,825 | 500 |
| Frequency/1000 cells | 2.9 | 2.6 | 0.5 | 6 |

**Supplementary Figures**

Figure 1. GTG-banded karyotype of case #20, a large white (Yorkshire) boar carrying the recurrent mos t(7;18)(q22,q11). Ideograms are placed on the left, normal chromosomes are placed in the middle, and their derivate chromosomes are placed on the right. Arrows indicate the presumed breakpoints. On the left, is the GTG-banded karyotype of the metaphase containing the mos t(7;18) in a Duroc boar. On the right, is a detailed presentation of the breakpoints on chromosome 7 and chromosome18 compared to the ideogram.


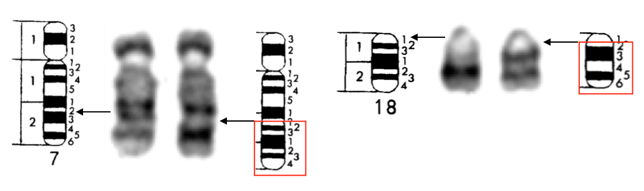

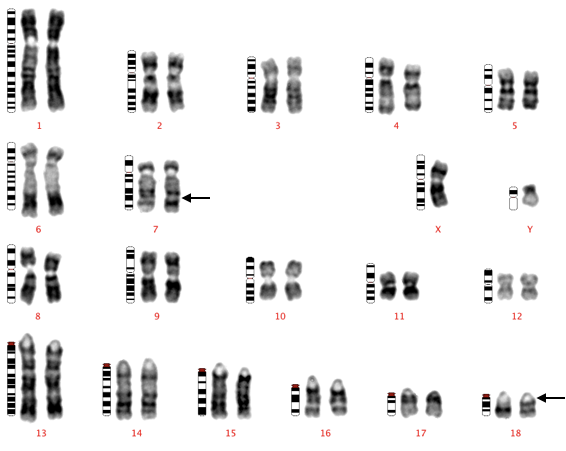


**Supplementary Figure 1. Case #20: mos t(7;18)(q22,q11)**

**Supplementary Figure 2. Case #21: mos t(9;18)(q22;q11)**


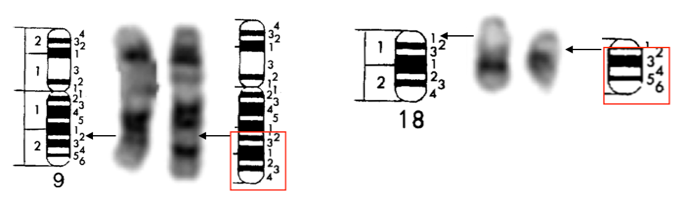

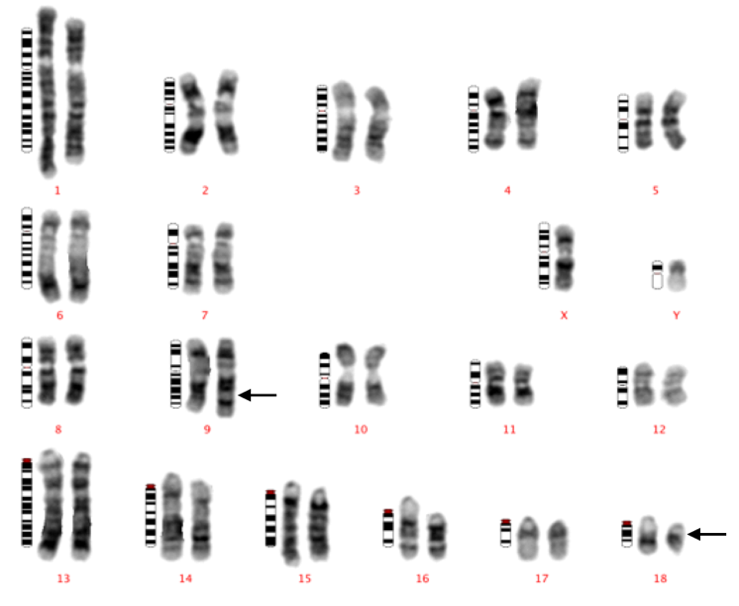


Figure 2. The GTG-banded karyotype of case #21, a Landrace boar carrying mos t(9;18)(q22;q11). Ideograms are placed on the left, normal chromosomes are placed in the middle, and their derivate chromosomes are placed on the right. Arrows indicate the presumed breakpoints on the derivative chromosomes. On the left, the complete chromosome profile of the abnormal cell. On the right, close-up picture of chromosome 9 and chromosome 18.

**Supplementary Figure 3. Case #38: mos t(9;18)(q22;q11)**


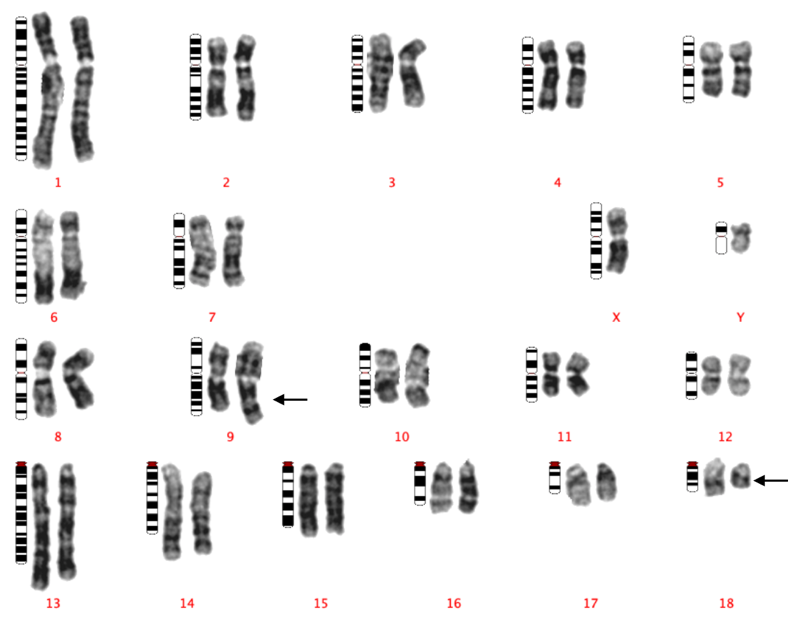

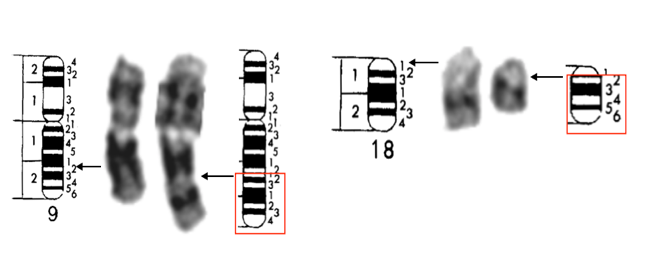


Figure 3. The GTG-banded karyotype of case #38, a Yorkshire boar carrying mos t(9;18)(q22;q11). Ideograms are placed on the left, normal chromosomes are placed in the middle, and their derivate chromosomes are placed on the right. Arrows indicate the presumed breakpoints on the derivative chromosomes. On the left, the complete chromosome profile of the abnormal cell. On the right, a close-up picture of chromosome 9 and chromosome 18 and their derivative chromosomes.

**Supplementary Figure 4. Case #16: mos t(7;9)(q24;q24), t(3;13)(q21;49)**


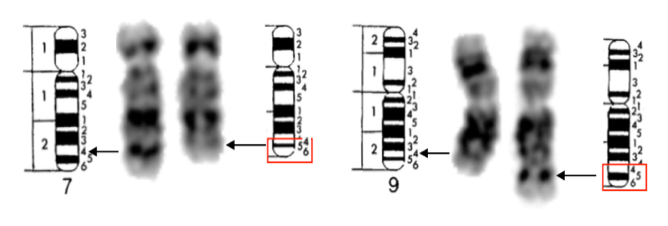


a.

A.

A.

A.

A.

A.

A.

A.

b.


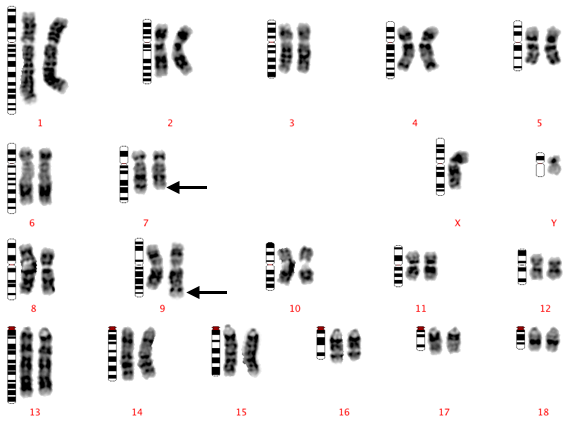

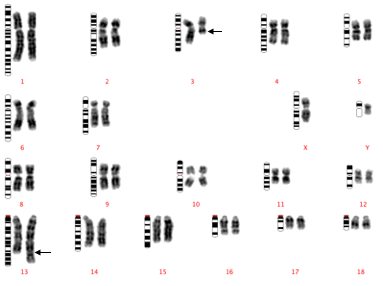


d.

c.

C.

C.

C.

Figure 11. GTG-banded karyotype of a Duroc boar carrying two mosaic translocations in different cells. Ideograms are placed on the left, normal chromosomes are placed in the middle, and their derivate chromosomes are placed on the right. Arrows indicate the presumed breakpoints*.* A. The GTG-banded karyotype of the metaphase containing the mos t(7;9). B. A detailed presentation of the breakpoints on SSC7 and SSC9 compared to the ideogram. C. GTG-banded karyotype of the abnormal metaphase mos t(3;13). D. Isolated SSC3 and SSC13 from the karyotype to showcase the breakpoints.C.

C.

C.

C.


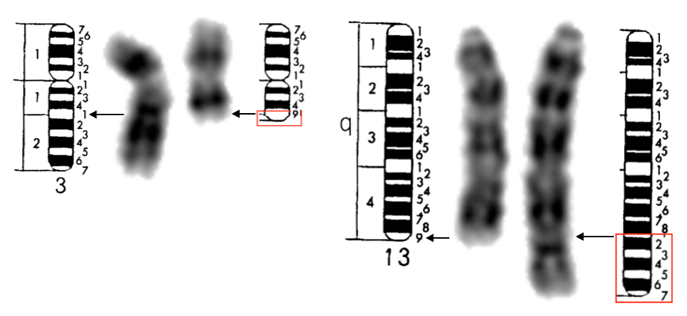


Figure 4. GTG-banded karyotype of case #16, a Duroc boar carrying two mosaic translocations in different cells. Ideograms are placed on the left, normal chromosomes are placed in the middle, and their derivate chromosomes are placed on the right. Arrows indicate the presumed breakpoints*.* (a) The GTG-banded karyotype of the metaphase containing the recurrent mos t(7;9). (b) A detailed presentation of the breakpoints on chromosome 7 and chromosome 9 compared to the ideogram. (c) GTG-banded karyotype of the abnormal metaphase mos t(3;13)(q21;49). (d) Isolated chromosome 3 and chromosome 13 from the karyotype to showcase the breakpoints.

**Supplementary Figure 5. Case #18: XX [42]/XY[7]/38, XY, t(7;9)(q24;q24)/38**


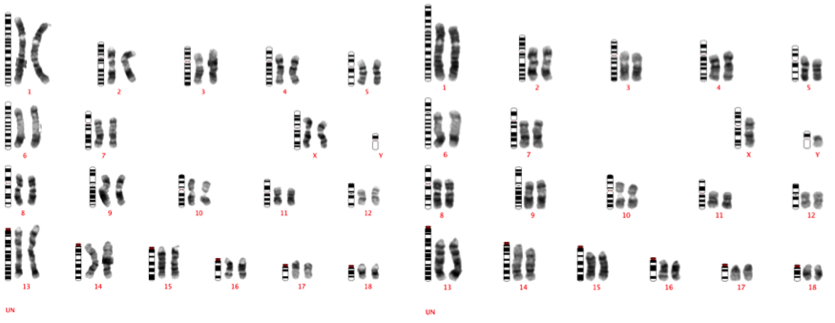


Figure 5. GTG-banded karyotype of case #18, a Landrace chimera boar with a chromosome makeup of 38, XX [42]/XY[7]/38, XY, t(7;9)(q24;q24)/38. Ideograms are placed on the left and normal chromosomes are placed on the right. On the left, the GTG-banded karyotype of the metaphase containing the XX cells. On the right is a metaphase representing the XY karyotype in the animal.

**Supplementary Figure 6. Case #17: mos t(7;9)(q24;q24), t(6;7)(q21,q22)**


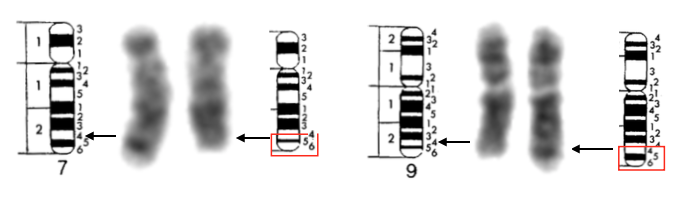


a.

A.

A.

A.

A.

A.

A.

A.

b.


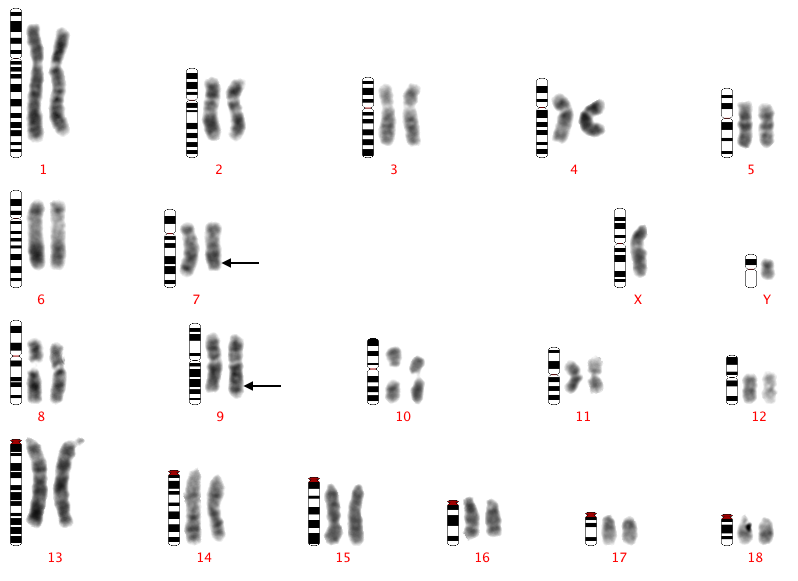

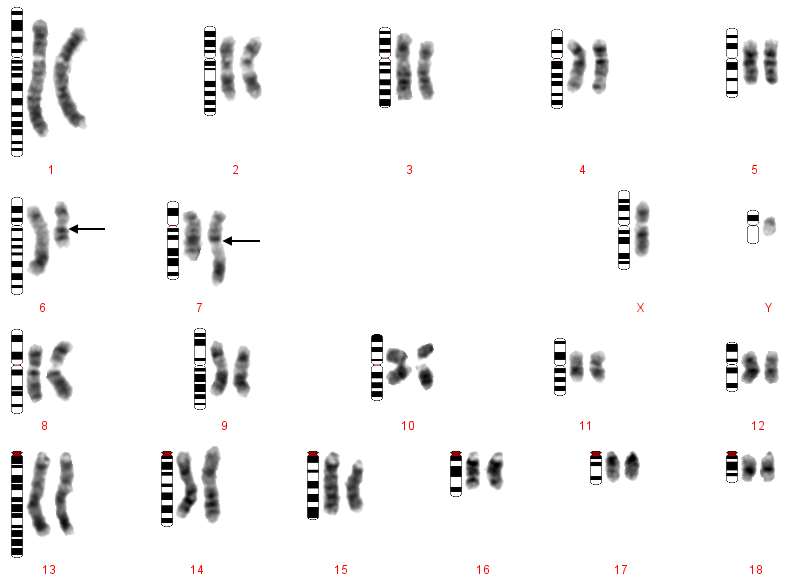


c.


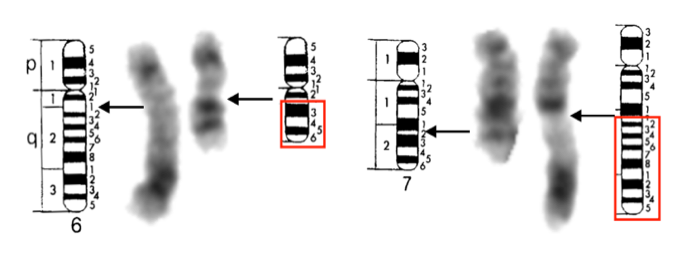


Figure 6. GTG-banded karyotype of case #17, a Yorkshire boar carrying two mosaic translocations in different cells. Ideograms are placed on the left, normal chromosomes are placed in the middle, and their derivate chromosomes are placed on the right. Arrows indicate the presumed breakpoints. (a) The GTG-banded karyotype of the metaphase containing the recurrent mos t(7;9). (b) A detailed presentation of the breakpoints on chromosome 7 and chromosome 9 compared to the ideogram. (c) GTG-banded karyotype of a the mos t(6;7)(q21,q22) observed in the second lymphocyte culture from the same animal. (d) Isolated chromosome 6 and chromosome 7 from the karyotype to showcase the breakpoints.

d.

**Supplementary Figure 7. Case #22: mos t(3;7)(p15;q13)**


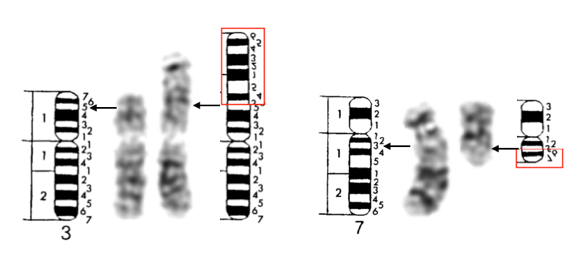

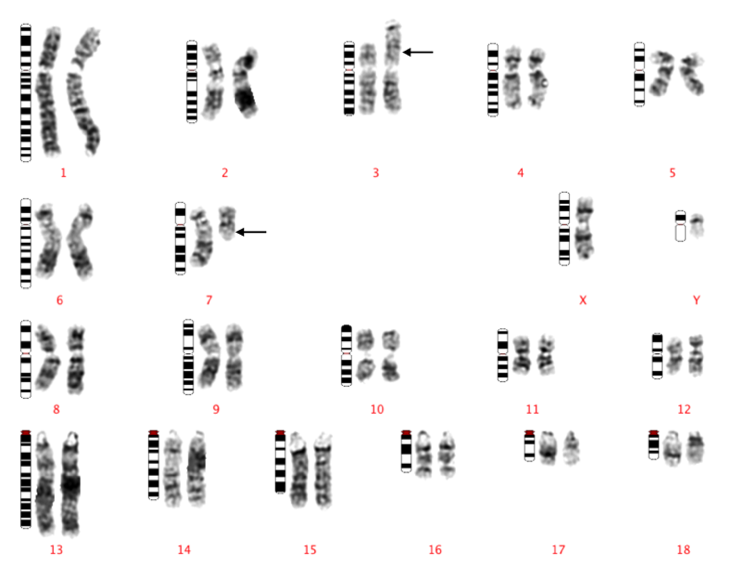


Figure 7. The GTG-banded karyotype of case #22, a Duroc boar carrying a mos t(3;7)(p15;q13). Ideograms are placed on the left, normal chromosomes are placed in the middle, and their derivate chromosomes are placed on the right. Arrows indicate the presumed breakpoints on the derivative chromosomes. On the left, the abnormal metaphase containing the t(3;7). On the right, a close up view of the approximate breakpoints involved in the reciprocal exchange.

**Supplementary Figure 8. Case #23: mos t(8;9)(p21;q24)**


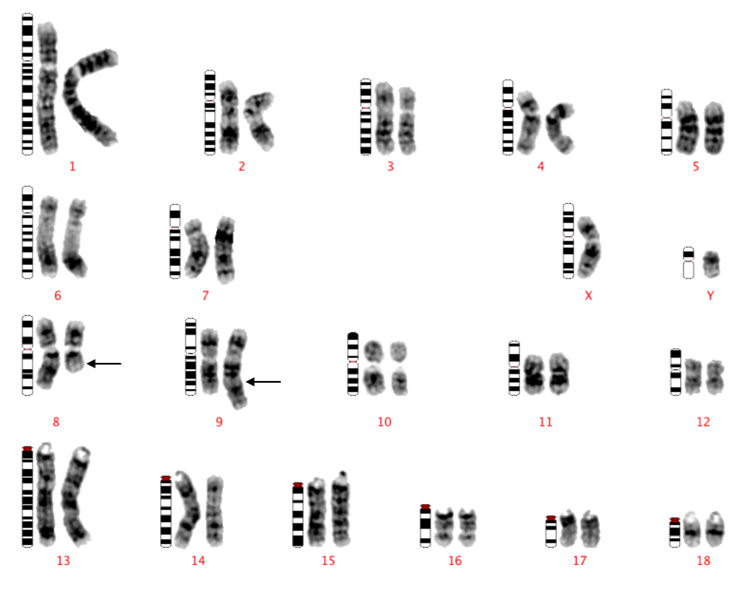

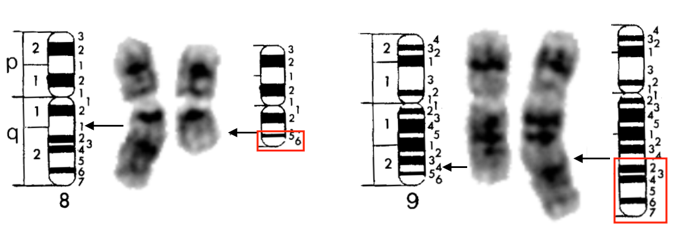


Figure 8. The GTG-banded karyotype of case #23, a Landrace boar carrying mos t(8;9)(p21;q24). Ideograms are placed on the left, normal chromosomes are placed in the middle, and their derivate chromosomes are placed on the right. Arrows indicate the presumed breakpoints on the derivative chromosomes. On the left, the abnormal metaphase with the translocation (8;9). On the right, close-up view of the approximate breakpoints involved in the reciprocal exchange.

**Supplementary Figure 9. Case #24: mos t(3;7)(q23;q26)**


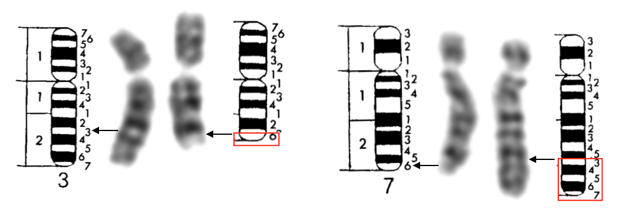

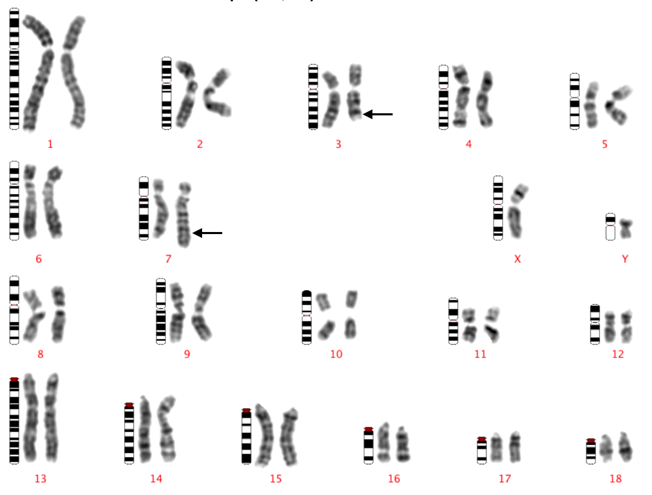


Figure 9. The GTG-banded karyotype of case #24, a Landrace boar carrying mos t(3;7)(q23;q26). Ideograms are placed on the left, normal chromosomes are placed in the middle, and their derivate chromosomes are placed on the right. Arrows indicate the presumed breakpoints on the derivative chromosomes. On the left, the karyotyped metaphase with the abnormal chromosome composition. On the right, an overview of the proposed breakpoints on the derivative chromosomes.

*.*

**Supplementary Figure 10. Case #25: mos t(3;10)(q23;p13)**


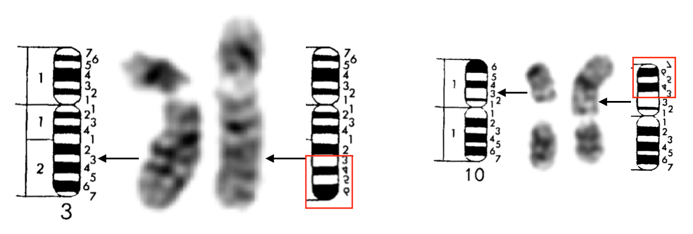

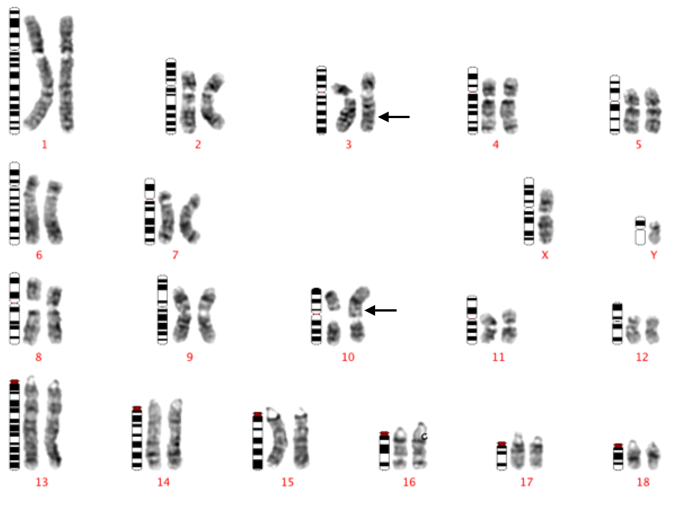


Figure 10. The GTG-banded karyotype of case #25, a Yorkshire boar carrying mos t(3;10)(q23;p13). Ideograms are placed on the left, normal chromosomes are placed in the middle, and their derivate chromosomes are placed on the right. On the left, the karyotype of mos t(3;10). On the right, a detailed description of breakpoints on each derivative chromosome.

**Supplementary Figure 11. Case #26: mos t(7;7)(q24;q15)**


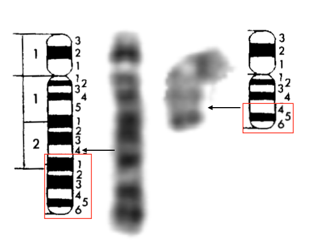

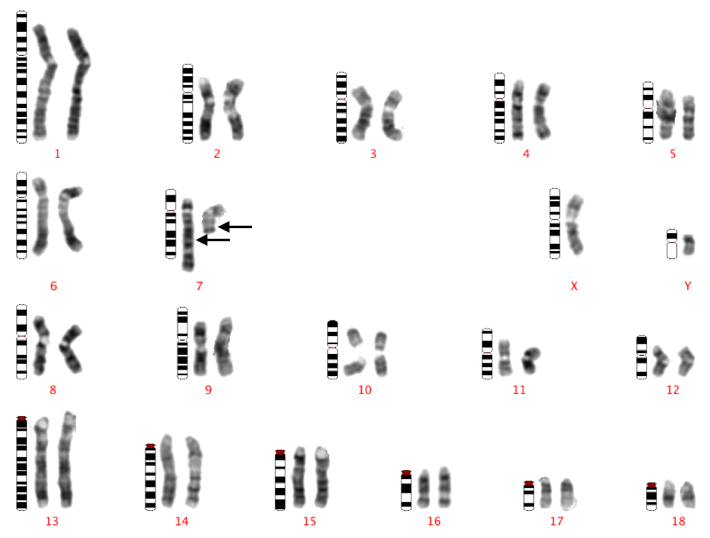


Figure 11. The GTG-banded karyotype of case #26, a Yorkshire boar carrying mos t(7;7)(q24;q15). Ideograms are placed on the left, normal chromosomes are placed in the middle, and their derivate chromosomes are placed on the right. Arrows indicate the presumed breakpoints on the derivative chromosomes. On the left, the complete chromosome profile of the abnormal t(7;7). On the right, the location of the presumed breakpoints on the chromosome 7 derivatives.

**Supplementary Figure 12. Case #27: mos t(7;9)(q26;q22)**


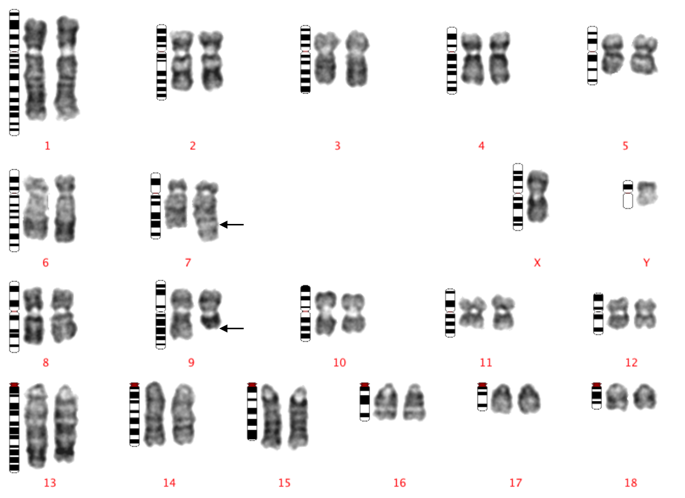

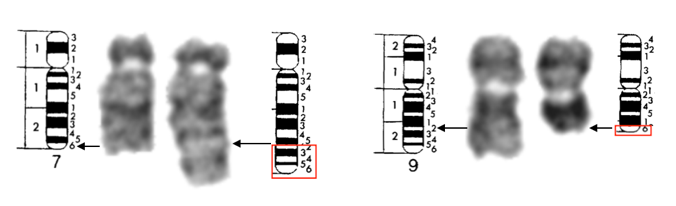


Figure 12. The GTG-banded karyotype of case #27, a Yorkshire boar carrying mos t(7;9)(q26;q22). Ideograms are placed on the left, normal chromosomes are placed in the middle, and their derivate chromosomes are placed on the right. Arrows indicate the presumed breakpoints on the derivative chromosomes. On the left, the chromosome profile of the t(7;9). On the right, the detailed description of the derivative chromosomes.

**Supplementary Figure 13. Case #28: mos t(2;8)(q23;q21)**


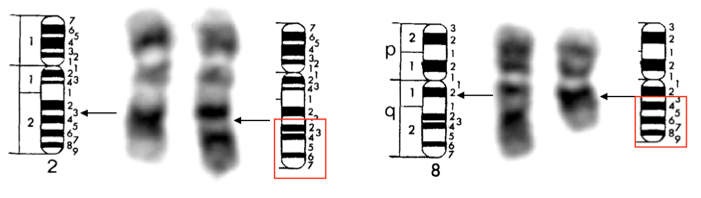

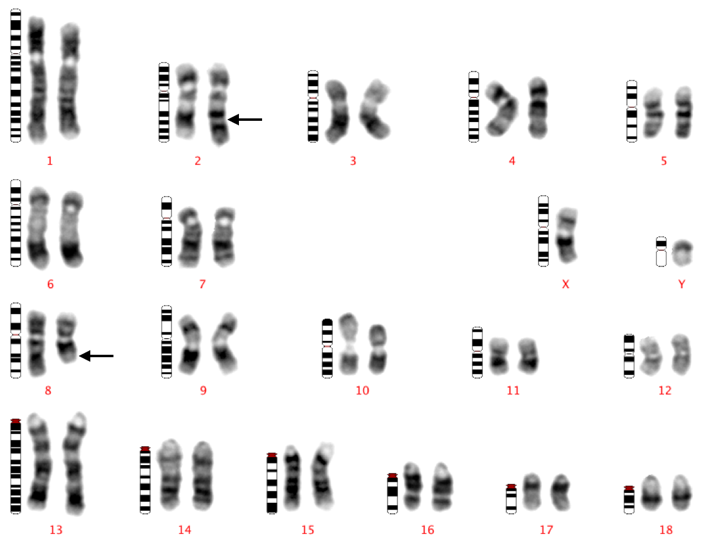


Figure 13. The GTG-banded karyotype of case #28, a Duroc boar carrying the mos t(2;8). Ideograms are placed on the left, normal chromosomes are placed in the middle, and their derivate chromosomes are placed on the right. Arrows indicate the presumed breakpoints on the derivative chromosomes. On the left, the complete chromosome profile of the abnormal cell. On the right, a detailed description of the presumed breakpoints.

**Supplementary Figure 14. Case #29: mos t(5;9)(q21;p22)**


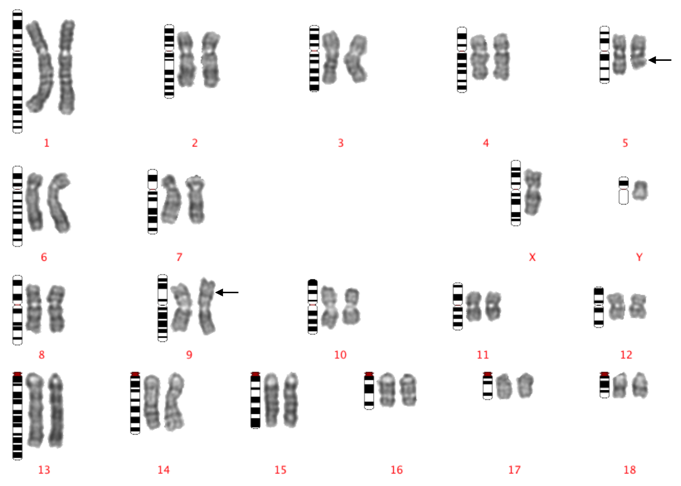

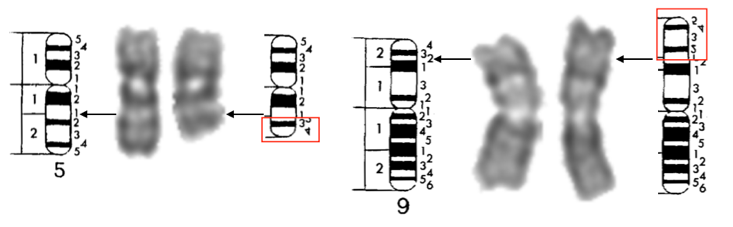


Figure 14. GTG-banded karyotype of case #29, a Duroc boar carrying mos t(5;9)(q21;p22). Ideograms are placed on the left, normal chromosomes are placed in the middle, and their derivate chromosomes are placed on the right. Arrows indicate the presumed breakpoints on the derivative chromosomes. On the left, the karyotype of the abnormal cell. On the right, the approximate breakpoints on chromosome 5 and chromosome 9 derivate chromosomes.

**Supplementary Figure 15. Case #30: mos t(6;16)(p15;q21)**


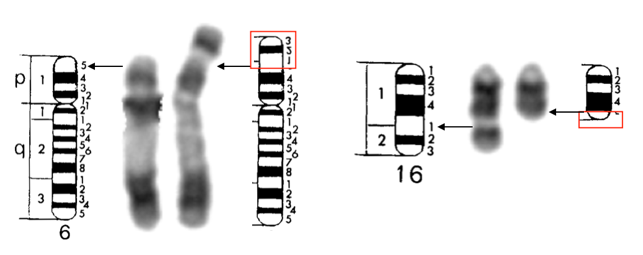

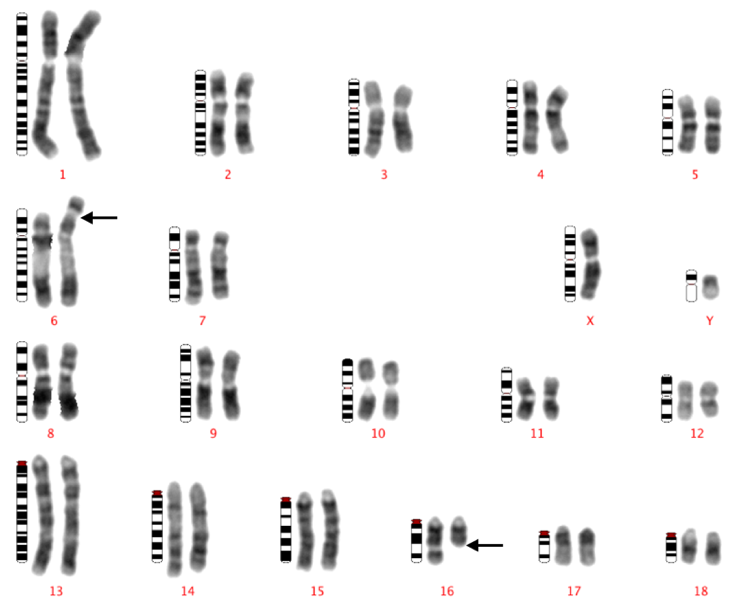


**Figure 15.** The GTG-banded karyotype of case #30, a Yorkshire boar carrying mos t(6;16)(p15;q21). Ideograms are placed on the left, normal chromosomes are placed in the middle, and their derivate chromosomes are placed on the right. Arrows indicate the presumed breakpoints on the derivative chromosomes. On the left, the karyotype of the abnormal cell. On the right, the detailed description of the approximate breakpoints on derivative chromosomes.

**Supplementary Figure 16. Case #31: mos t(7;9)(q15;q15)**


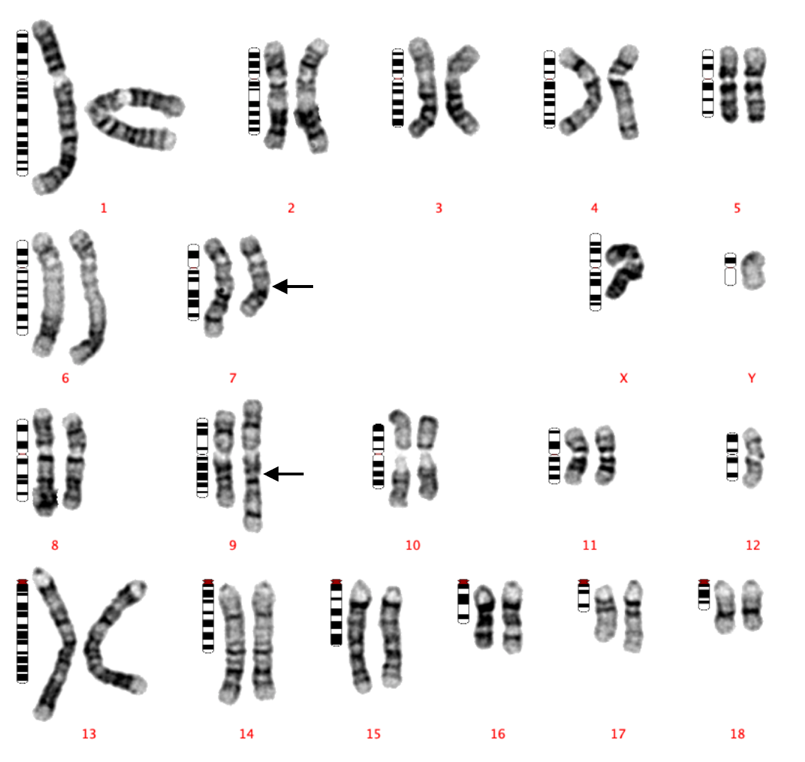

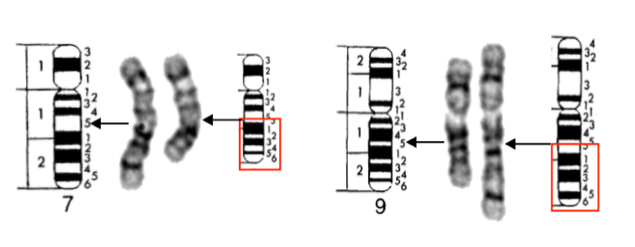


Figure 16. The GTG-banded karyotype of case #31, a Duroc boar carrying mos t(7;9) (q15;q15). Ideograms are placed on the left, normal chromosomes are placed in the middle, and their derivate chromosomes are placed on the right. Arrows indicate the presumed breakpoints on the derivative chromosomes. On the left, the karyotype of the abnormal cell is represented. On the right, the presumed breakpoints on the derivate chromosome 7 and chromosome 9 are illustrated.

**Supplementary Figure 17. Case #32: mos t(7;13)(q22;q21)**


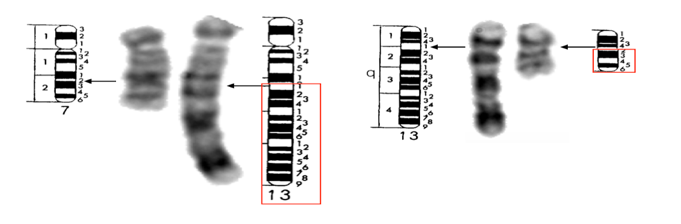

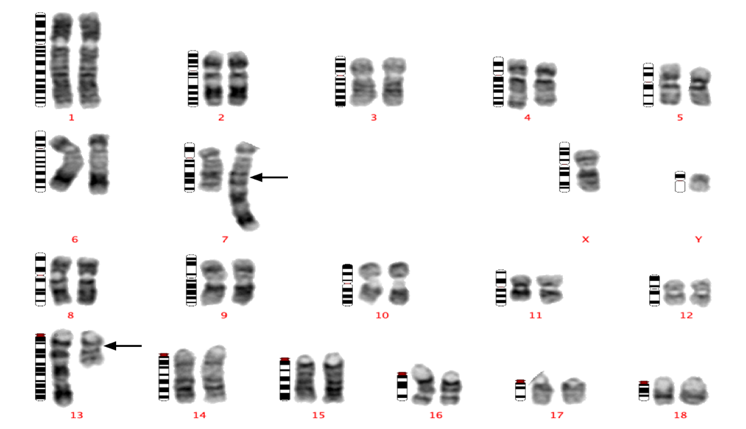


Figure 17. The GTG-banded karyotype of case #32, a Duroc boar carrying mos t(7;13)(q22;q21). Ideograms are placed on the left, normal chromosomes are placed in the middle, and their derivate chromosomes are placed on the right. Arrows indicate the presumed breakpoints on the derivative chromosomes. On the left, the karyotype of the abnormal cell. On the right, the presumed breakpoints on the derivate chromosome 7 and chromosome 13.

**Supplementary Figure 18. Case #36: mos t(7;9)(q24;q24)**


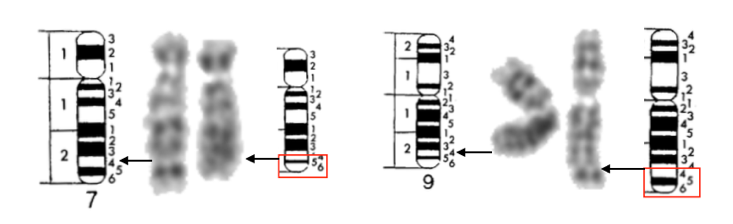

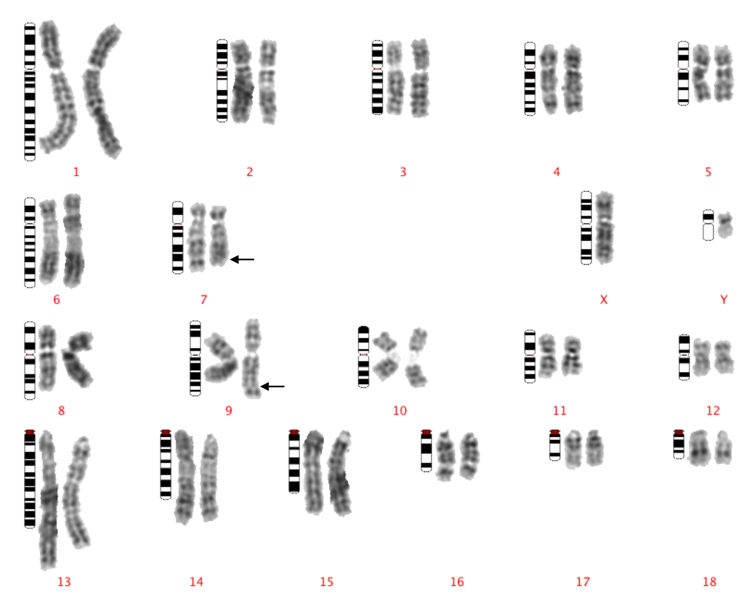


Figure 18. GTG-banded karyotype of case #36, a Duroc boar carrying the recurrent mos t(7;9). Ideograms are placed on the left, normal chromosomes are placed in the middle, and their derivate chromosomes are placed on the right. Arrows indicate the presumed breakpoints. On the left, the GTG-banded karyotype of the metaphase containing the mos t(7;9). On the right, a detailed presentation of the breakpoints on SSC7 and SSC9 compared to the ideogram.

**Supplementary Figure 19. Case #37: mos t(10;12)(q15;q11)**


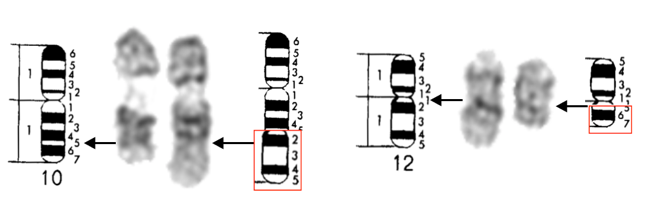

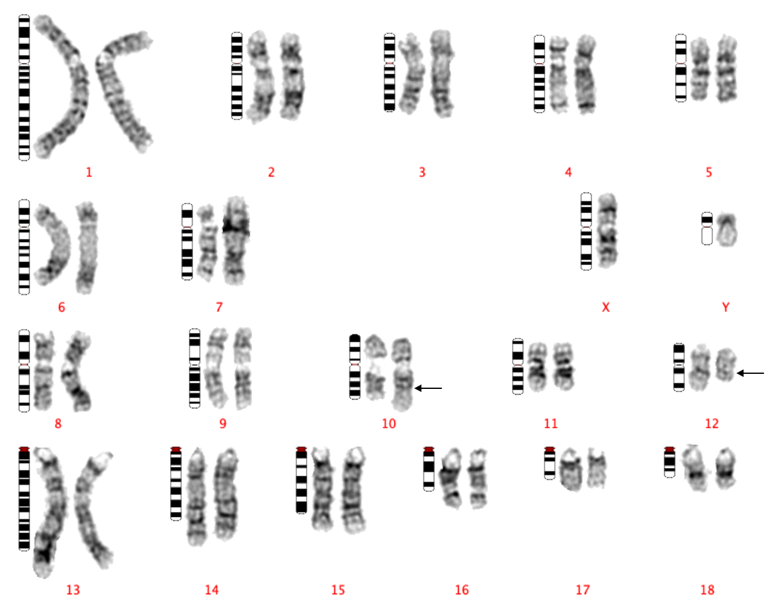


Figure 19. GTG-banded karyotype of case #37, a Duroc boar carrying mos t(10;12)(q15;q11) in the culture induce by PWM. Ideograms are placed on the left, normal chromosomes are placed in the middle, and their derivate chromosomes are placed on the right. Arrows indicate the presumed breakpoints*.* On the left, the GTG-banded karyotype of the metaphase containing the mos t(10;12). On the right, a detailed presentation of the breakpoints on chromosome 10 and chromosome 12 compared to the ideogram.

**Supplementary Figure 20. Case #39: mos t(5;7)(q21;q22)**


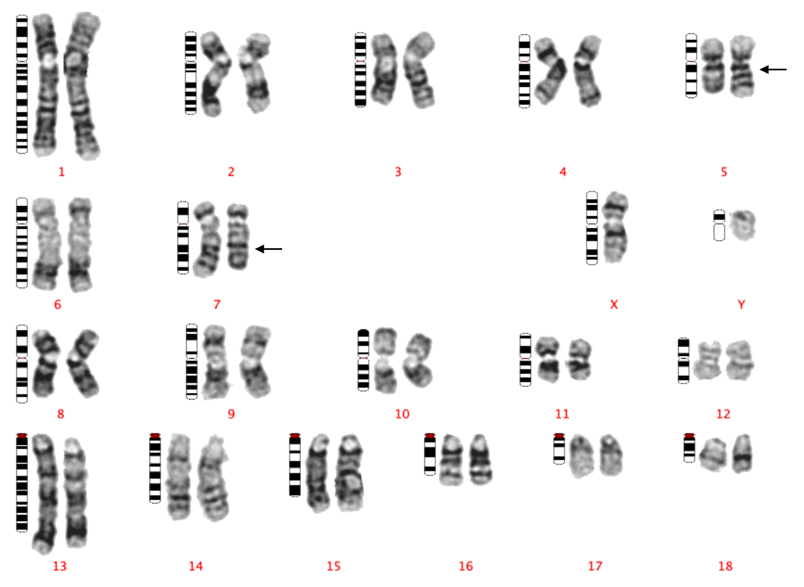

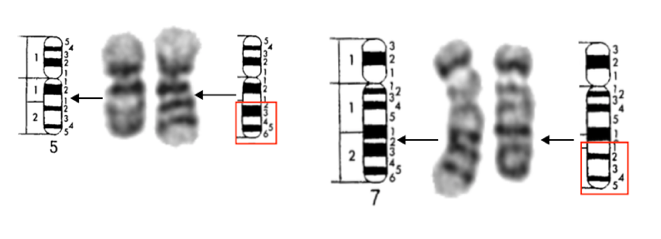


Figure 20. The GTG-banded karyotype of case #39, a Yorkshire boar carrying mos t(5;7)(q21;q22) identified in the PWM culture. Ideograms are placed on the left, normal chromosomes are placed in the middle, and their derivate chromosomes are placed on the right. Arrows indicate the presumed break points on the derivative chromosomes. On the left, the complete chromosome profile of the abnormal cell. On the right, a close-up picture of chromosome 5 and chromosome 7.

**Supplementary Figure 21. Case #40: Abnormal metaphase in fibroblast chromosomes**


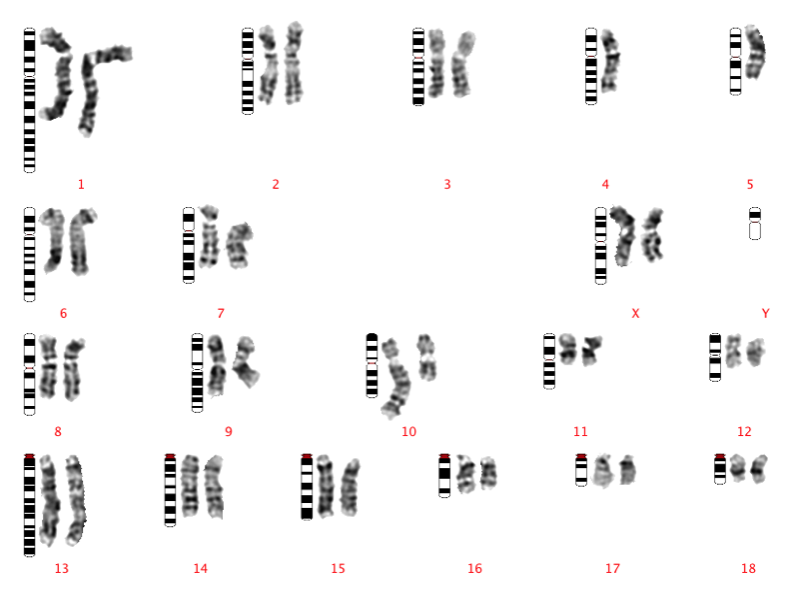


Figure 21. A metaphase containing an abnormal karyotype. Due to the incomplete chromosome count a delineation of the abnormality was not possible.
